# Inactivation of bone morphogenetic protein 2 may predict clinical outcome and poor overall survival for renal cell carcinoma through epigenetic pathways

**DOI:** 10.18632/oncotarget.3445

**Published:** 2015-03-07

**Authors:** Yozo Mitsui, Hiroshi Hirata, Naoko Arichi, Miho Hiraki, Hiroaki Yasumoto, Inik Chang, Shinichiro Fukuhara, Soichiro Yamamura, Varahram Shahryari, Guoren Deng, Sharanjot Saini, Shahana Majid, Rajvir Dahiya, Yuichiro Tanaka, Hiroaki Shiina

**Affiliations:** ^1^ Department of Urology, Shimane University Faculty of Medicine, Enya-cho, Izumo, Japan; ^2^ Department of Urology, San Francisco Veterans Affairs Medical Center and University of California San Francisco, San Francisco, California, USA; ^3^ Department of Oral Biology, Yonsei University College of Densitry, Seoul, South Korea; ^4^ Department of Urology, Osaka University Graduate School of Medicine, Suita, Japan

**Keywords:** bone morphogenetic protein 2, renal cell carcinoma, DNA methylation, molecular marker

## Abstract

We investigated whether impaired regulation of bone morphogenetic protein-2 (BMP-2) via epigenetic pathways is associated with renal cell carcinoma (RCC) pathogenesis. Expression and CpG methylation of the *BMP-2* gene were analyzed using RCC cell lines, and 96 matched RCC and normal renal tissues. We also performed functional analysis using BMP-2 restored RCC cells. A significant association of *BMP-2* mRNA expression was also found with advanced tumor stage and lymph node involvement, while lower *BMP-2* mRNA expression was significantly associated with poor overall survival after radical nephrectomy. In RCC cells, BMP-2 restoration significantly inhibited cell proliferation, migration, invasion, and colony formation. In addition, BMP-2 overexpression induced *p21^WAF1/CIP1^* and *p27^KIP1^* expression, and cellular apoptosis in RCC cells. *BMP-2* mRNA expression was significantly enhanced in RCC cells by 5-aza-2′-deoxycitidine treatment. The prevalence of *BMP-2* promoter methylation was significantly greater and *BMP-2* mRNA expression was significantly lower in RCC samples as compared to normal kidney samples. Furthermore, a significant correlation was found between *BMP-2* promoter methylation and mRNA transcription in tumors. Aberrant *BMP-2* methylation and the resultant loss of BMP-2 expression may be a useful molecular marker for designing improved diagnostic and therapeutic strategies for RCC.

## INTRODUCTION

Renal cell carcinoma (RCC) is one of the most commonly encountered urological cancers, accounting for 2-3% of all tumor malignancies in adults [1]. The major cause of death from RCC is metastasis and the 5-year survival rate for affected patients with metastasis is less than 10% [2]. Indeed, 25% of RCC patients show metastasis at the time of diagnosis [3]. In addition, up to one third of localized RCC patients experience recurrence and/or metastasis after curative radical surgery [4]. Although several prognostic models of RCC based on clinical parameters have been developed [5, 6], they all lack accuracy, probably due to the biologically heterogeneous nature of the disease. Therefore, efficient and reliable molecular biomarkers to predict cancer progression are urgently needed to develop better therapeutic and diagnostic strategies.

Bone morphogenetic protein 2 (BMP-2), a member of the transforming growth factor (TGF)-β superfamily, has potent activities to induce the entire cascade of cartilage and ectopic osteogenesis [7]. In addition to bone formation, BMP-2 plays important roles in cell differentiation, proliferation, morphogenesis, and apoptosis [8-11]. Similar to TGF-β, BMP-2 exerts its effect via 2 types of transmembrane serine/threonine kinase receptors; BMP receptor type I (BMPRI) and II (BMPRII). BMP-2 binding to BMPRII triggers phosphorylation of BMPRI, and activation of downstream signaling via Sma- and Mad-related (Smad) proteins; e.g., Smad1, Smad5, and Smad8. Phosphorylated Smad1/5/8 subsequently form complexes with Smad4 and then translocates to the nucleus to regulate a variety of genes that arrest cell growth and induce apoptosis [12, 13].

*BMP-2* is thought to be a putative tumor-suppressor gene in several types of cancer (i.e., gastric, colon, prostate, adrenal) [10, 11, 14-17]. Recently, Wang et al. [18] demonstrated that BMP-2 inhibits RCC growth by causing cell cycle arrest in the G1 phase. On the other hand, Markić et al. [19] showed that expression levels of BMP-2 were strongly elevated with increased TNM stage in clinical RCC. However, the biological effects of BMP-2 on RCC development and progression remain to be fully elucidated, because only limited information is available for BMP-2 in human RCC.

DNA methylation of CpG islands involving the promoter of tumor suppressor genes is a well-known mechanism underlying gene silencing, which leads to functional loss as a tumor suppressor [20, 21]. Previous studies have shown that the expression level of BMP-2 is frequently down-regulated because of promoter CpG hypermethylation [14, 15]. Therefore, we hypothesized that impaired regulation of BMP-2 via an epigenetic pathway may be associated with RCC pathogenesis.

In the present study, we assessed the correlation between expression of the *BMP-2* gene and epigenetic mechanisms using 2 RCC cells lines, as well as 96 matched RCC and normal renal tissues. We also evaluated the association of BMP-2 expression and BMP-2 CpG methylation status with clinical parameters and prognosis in cases of RCC following radical nephrectomy. Finally, we over-expressed BMP-2 in kidney cancer cells and performed functional analyses.

## RESULTS

### BMP-2 is down-regulated in RCC cell lines and RCC tissues

**Figure 1:**
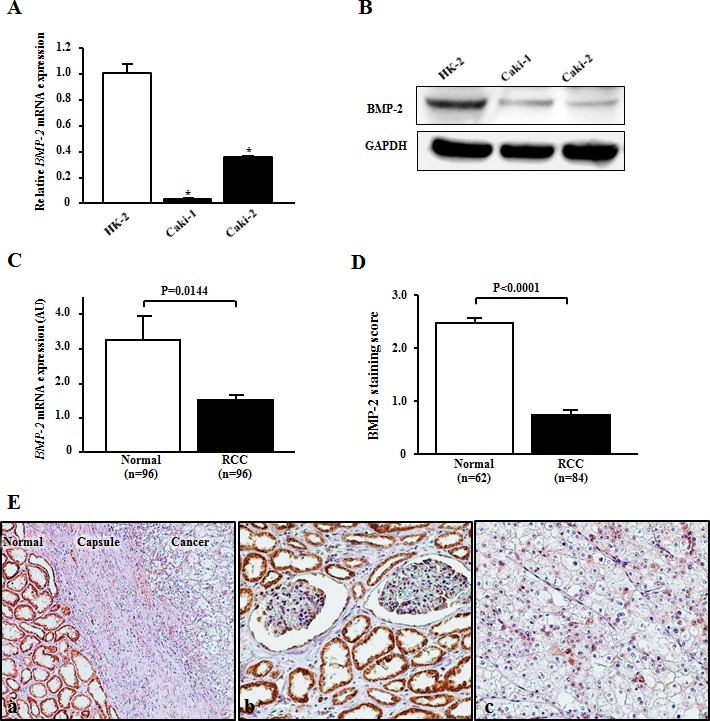
BMP-2 expression in RCC cell lines and tissues (A) Relative *BMP-2* mRNA expression levels in RCC cell lines (Caki-1 and Caki-2) and normal kidney cells (HK-2). The expression level of *BMP-2* mRNA was significantly down-regulated in Caki-2 and Caki-2 cells. *P<0.0001. (B) Representative immunoblotting image displaying BMP-2 expression in HK-2, Caki-1, and Caki-2 cells. BMP-2 protein was down-regulated in RCC as compared to HK-2 cells. (C) Expression of *BMP-2* mRNA in clinical samples. RCC samples showed a lower level of *BMP-2* mRNA expression in comparison with normal renal tissues (p=0.0144). (D) BMP-2 staining scores of clinical samples. BMP-2 protein expression in RCC samples was significantly lower as compared to normal kidney tissues (P<0.0001). (E) Representative immunostaining of BMP-2 in a clinical sample. (a) Strong BMP-2 staining was more common in the adjacent normal cells than in the cancer cells (×100). (b) Strong cytoplasmic and/or nuclear staining of BMP-2 was observed in normal renal tubules (×200). (c) Weak cytoplasmic staining of BMP-2 was observed in RCC tumors (×200).

To determine *BMP-2* mRNA and protein expression, RT-PCR and Western blotting analyses were performed using HK-2, Caki-1, and Caki-2 cells. Both *BMP-2* mRNA (Fig. 1A) and protein expression (Fig. 1B) were significantly down-regulated in the RCC cell lines as compared with the nonmalignant HK-2 cells. Next, BMP-2 expression was evaluated in 96 RCC samples and matched normal renal tissues. As shown in Fig. 1C, RCC showed a lower level of *BMP-2* mRNA expression in comparison with that of the corresponding normal renal tissues (P=0.0144). We also investigated the expression of BMP-2 using immunohistochemical staining. BMP-2 was significantly higher in the tubular cytoplasm of normal renal cells as compared to that of the RCC (P<0.0001; Fig. 1D, E). Furthermore, there was a positive correlation between BMP-2 mRNA transcription and protein level (data not shown).

### BMP-2 is regulated by promoter CpG methylation in RCC

**Figure 2:**
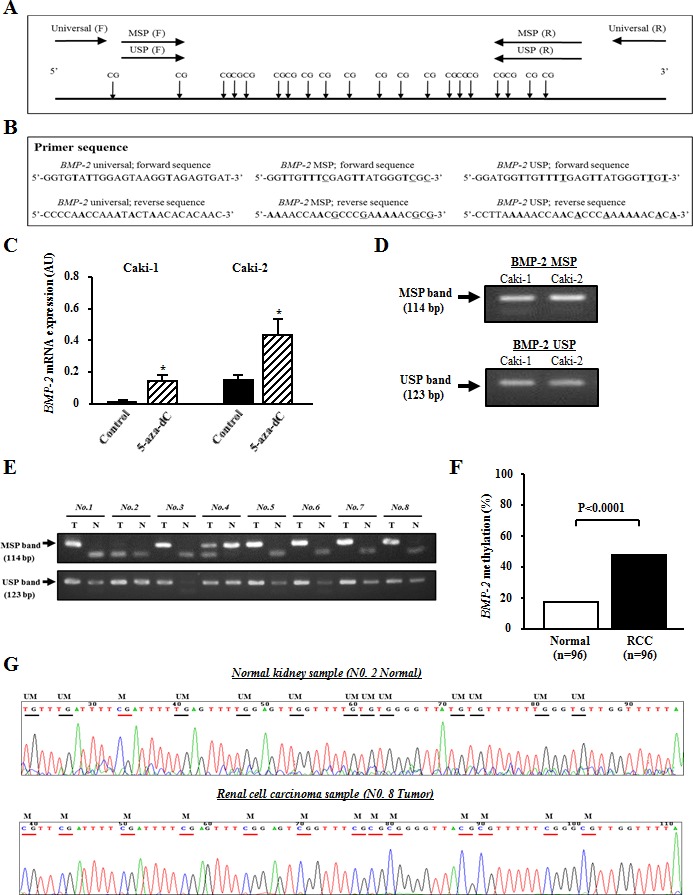
Analysis of *BMP-2* methylation in RCC cell lines and clinical samples (A) Schema of *BMP-2* promoter and locations of the primers. The universal primers (F, forward; R, reverse) used did not contain any CpG sites within the primer sequence. The F and R methylation-specific PCR (MSP) primers contained 3 and 4 CpG sites, respectively, with un-methylation-specific PCR (USP) primers designed in the same manner. (B) Primer sequences. Boldface indicates difference with modified sequence, and underline indicates changes between methylated and un-methylated sequences after bisulfite modification. (C) Alteration of *BMP-2* expression before and after de-methylation. In both Caki-1 and Caki-2 cell lines, the expression level of the mRNA transcript of *BMP-2* was significantly increased after 5-aza-dC treatment as compared with that before de-methylation. *P<0.0001. (D) MSP and USP bands in Caki-1 and Caki-2 cells, which were partially methylated. (E) Representative results of MSP and USP of the *BMP-2* promoter in clinical samples. Top and bottom show MSP and USP bands, respectively, from the same samples. (F) *BMP-2* methylation status in clinical samples. The prevalence of *BMP-2* methylation was significantly higher in RCC samples than normal renal tissues (P<0.0001). (G) Typical bisulfite DNA sequencing in normal kidney and RCC samples. In the normal kidney samples, the majority of cytosines within the CpG sites were completely converted to thymines after bisulfite modification, whereas in the RCC samples, the majority of cytosines remained virtually unchanged after bisulfite modification. Horizontal bar, CpG sites; UM, un-methylation; M, methylation.

We used 5-aza-dC to screen for the epigenetic status of *BMP-2* in RCC cell lines. In Caki-1 and Caki-2 cells, the expression level of the *BMP-2* mRNA transcript was significantly increased after 5-aza-dC treatment (Fig. 2C), suggesting that promoter CpG methylation may be associated with *BMP-2* expression in these cells. To confirm the relationship between CpG methylation and expression of the *BMP-2* mRNA transcript, we performed MSP analysis. As shown in Fig. 2A and B, MSP and USP primers were designed based on a previous report [14]. Caki-1 and Caki-2 cells, which slightly express the *BMP-2* gene, were partially methylated (Fig. 2D).

We further performed MSP analysis of the 96 RCC tissue samples. Representative MSP and USP bands of 8 matched RCC and normal renal tissues are shown in Fig. 2E. Most RCC tissues showed both MSP and USP bands, whereas most normal renal tissues showed only a USP band. Forty-six of the 96 RCC tissues (47.9%) were found to be positive for *BMP-2* methylation, while 16 of 96 normal kidney tissues (17.7%) were positive (P<0.0001; Fig. 2F). Bisulfite DNA sequencing was also performed to confirm whether the MSP bands reflected the true methylation status of the CpG sites. Representative bisulfite DNA sequencing findings for RCC and normal renal tissues are shown in Fig. 2G. In a normal kidney sample (*No. 2 Normal*), the majority of cytosines within CpG sites were completely converted to thymines (un-methylated) after bisulfite modification. On the other hand, the majority of cytosines remained virtually unchanged after bisulfite modification in an RCC sample (*No. 8 Tumor*). These results indicated that methylation analysis using a combination of MSP and USP was consistent with the results from bisulfite DNA sequencing.

**Figure 3:**
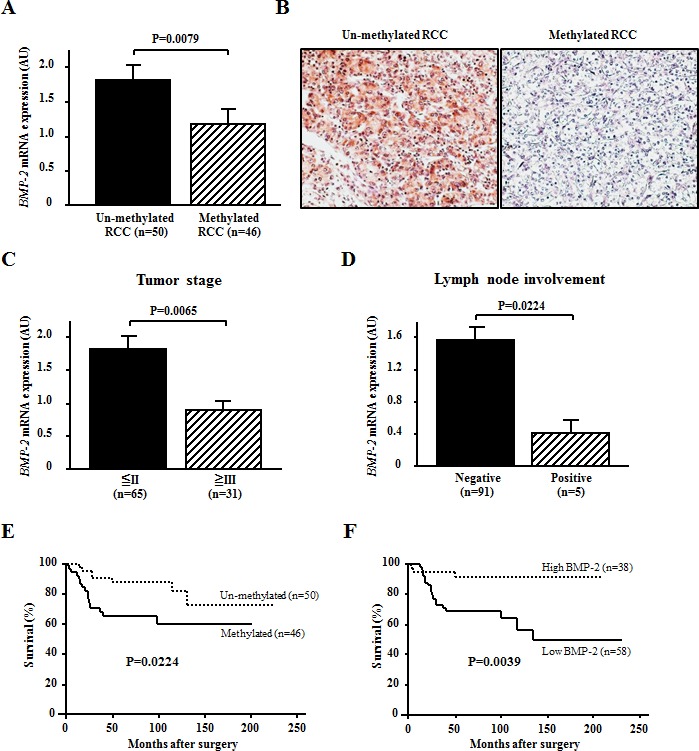
Effects of BMP-2 methylation status on the expression level of BMP-2 mRNA and association with clinicopathological findings (A) Relationship between *BMP-2* methylation status and *BMP-2* mRNA expression. In RCC samples, *BMP-2* methylation (+) resulted in a significantly lower level of expression of the *BMP-2* mRNA transcript as compared to *BMP-2* un-methylation (−) (p=0.0079). (B) Representative immunostaining of BMP-2 in RCC samples. Un-methylated RCC cells exhibited positive staining (×200). Methylated RCC cells exhibited negative staining (×200). (C, D) Relationships of *BMP-2* expression with clinicopathological findings. A lower level of expression of the *BMP-2* mRNA transcript was significantly associated with a higher prevalence of cases with advanced disease (greater than III) (p=0.0065) and lymph node involvement (p=0.0224). (E, F) Kaplan-Meier curves for overall survival after radical nephrectomy. Both methylation (+) and lower *BMP-2* expression were significantly associated with poor prognosis (P=0.0224, P=0.0039, respectively).

Next, we evaluated the correlation between methylation status and *BMP-2* expression in RCC samples. A significant inverse correlation was found between *BMP-2* mRNA transcripts and methylation of the *BMP-2* promoter in the RCC samples (P=0.0079; Fig. 3A). In addition, RCC samples with an un-methylated alle of *BMP-2* exhibited positive staining, while methylated RCC samples exhibited negative staining (Fig. 3B). Thus, expression of *BMP-2* may be silenced via promoter CpG methylation in RCC.

### *BMP-2* methylation and resultant loss of BMP-2 correlated with poor prognosis in RCC

Clinicopathological findings of the 96 RCC patients with *BMP-2* methylation status are shown in Table 1. *BMP-2* methylation was found to have a significant association with infiltrative growth pattern and systematic metastasis (P=0.038, P=0.0024, respectively). Also, the RCC patients with positive methylation had a higher stage as compared to those with negative methylation, though the difference did not reach statistical significance (P=0.0701). However, there was no significant association of *BMP-2* methylation with tumor grade found. Correlations of clinicopathological findings with *BMP-2* mRNA expression are shown in Fig. 3C and D. A lower expression level of the *BMP-2* mRNA transcript was also significantly correlated with high-stage disease and lymph node involvement (P=0.0065, P=0.024, respectively).

**Table 1:** BMP-2 methylation in relation to clinicopathological findings

variables	*BMP-2* methylation		p value
	Positive N=46	Negative N=50	
Median age (yrs)	66	62	0.1190
Gender			
male	28(60.9%)	35(70.0%)	0.3467
female	18(39.1%)	15(30.0%)	
Stage(Robson's classification)			0.0701
less than II	27(58.7%)	38(76.0%)	
more than III	19(41.3%)	12(24.0%)	
Tumor grade			0.1397
less than 2	39(84.8%)	47(94.0%)	
more than 3	7(15.2%)	3(6.0%)	
Infiltrative growth pattern (inf)			0.0380
alpha	20(43.5%)	31(62.0%)	
beta or gamma	25(54.3%)	16(32.0%)	
unknown	1(2.2%)	3(6.0%)	
Systematic metastasis			0.0024
positive	10(21.7%)	1(2.0%)	
negative	36(78.3%)	49(98.0%)	

Kaplan-Meier curves for overall survival (OS) after radical nephrectomy are shown in Fig. 3E and F. OS in the *BMP-2* methylated group was significantly worse in comparison with that in the *BMP-2* un-methylated group (P=0.025; Fig. 3E). Likewise, when the expression level of the *BMP-2* mRNA transcript was divided into 2 groups based on mean values, lower expression was significantly associated with poor prognosis (P=0.039; Fig. 3F). As shown in Table 2, *BMP-2* methylation status, *BMP-2* mRNA expression, tumor stage, and tumor grade were identified as significant factors contributing to OS in univariate analysis. When these variables were subjected to a multivariate model, *BMP-2* expression and tumor stage were shown to be significantly independent predictors for OS after a radical nephrectomy.

**Table 2:** Uni- and multivariate analyses for predicting over-all survival rate

Variables	Univariate analysis		Multivariate analysis	
	Hazard ratio	P value	Hazard ratio	P value
	(95% confience interval)		(95% confience interval)	
Age (years)	1.000 (0.965-1.037)	0.9845	―	―
Gender (male vs. female)	0.690 (0.268-1.778)	0.4421	―	―
*BMP-2* methylation status (positive vs. negative)	0.363 (0.146-0.901)	0.0289	0.631 (0.245-1.624)	0.3393
*BMP-2* expression (higher vs. lower)	5.064 (1.487-17.247)	0.0095	3.964 (1.074-14.634)	0.0387
Tumor stage (I or II vs. III or greater)	0.194 (0.078-0.483)	0.0004	0.295 (0.110-0.791)	0.0153
Tumor grade (1 or 2 vs. 3)	0.317 (0.106-0.946)	0.0394	0.537 (0.158-1.831)	0.3207

### BMP-2 inhibits renal cancer cell viability, migration, invasion, and colony-formation

To determine the functional significance of BMP-2 in RCC, we examined whether over-expression of *BMP-2* has effects on cell viability, migration and invasion properties, and colony-formation ability of RCC cell lines. Initially, mRNA expression of the *BMP-2* receptors *BMPRIa, BMPRIb*, and *BMPRII* was evaluated, because BMP-2 induces a physiological response via their activation [12, 13]. As shown in Supplementary Figure S1, all of the BMP receptors were found to be expressed in Caki-1 and Caki-2 cells, consistent with previous findings [18]. Our result indicates that RCC cells have a potential capability of being activated by BMP-2, though they have lower levels of *BMPRII* as compared to HK-2 cells.

**Figure 4:**
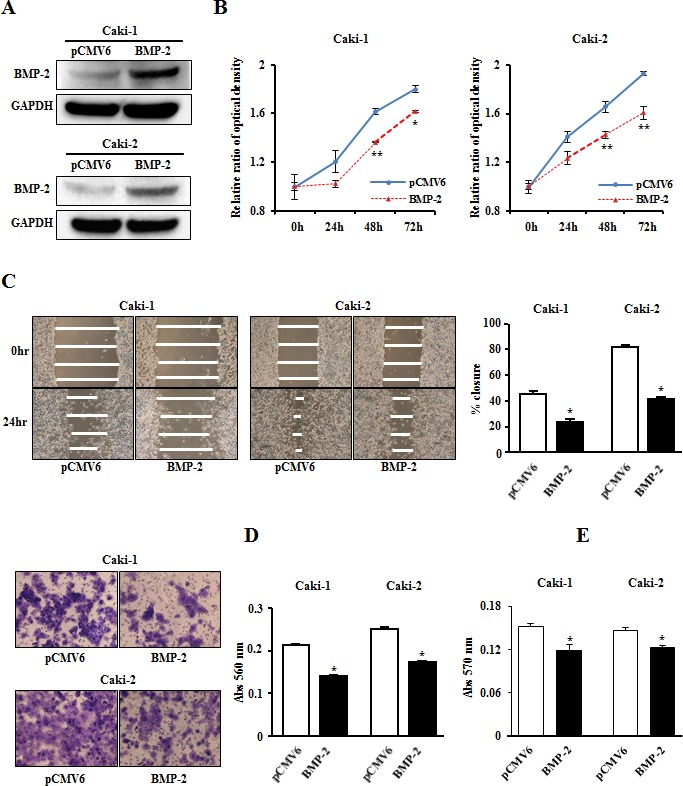
Effects of BMP-2 overexpression on cell proliferation, migration, invasion, and colony-formation (A) BMP-2 expression level in RCC cell lines (Caki-1, Caki-2) were determined by immunoblotting analysis at 48 hours after transfection of a plasmid containing human BMP-2. (B) Cell viability was analyzed using an MTS cell proliferation assay at 24, 48, 72, and 96 hours after transient transfection. Overexpression of BMP-2 significantly inhibited cell viability. *P<0.05, **P<0.01. (C) Representative images from wound healing assay. After transfection (48 hours), a wound was formed by scraping, then measured 24 hours later. Over-expression of BMP-2 significantly inhibited cell migration. *P<0.0001. (D) Representative images from invasion assay. Overexpression of BMP-2 resulted in significantly decreased cell invasion. *P<0.0001. (E) Overexpression of BMP-2 significantly inhibited colony formation ability. *P<0.01.

After transient transfection of a plasmid containing human BMP-2 into Caki-1 and Caki-2 cells, significant amounts of BMP-2 protein were detected by Western blotting (Fig. 4A). Cell proliferation (Fig. 4B) and wound healing (Fig. 4C) results demonstrated significant inhibition with BMP-2 transfectants for both Caki-1 and Caki-2 cells as compared to the control vector transfectants. Matrigel invasion assay also showed that the number of invaded cells was significantly decreased in the BMP-2 transfectants as compared with their control counterparts (Fig. 4D). In addition, overexpression of BMP-2 inhibited colony-formation in both Caki-1 and Caki-2 cells (Fig. 4E). Thus, these results suggest that BMP-2 plays an important role in RCC progression.

### BMP-2 induces p21^WAF1/CIP1^ and p27^KIP1^ expression in RCC via the Smad pathway

**Figure 5:**
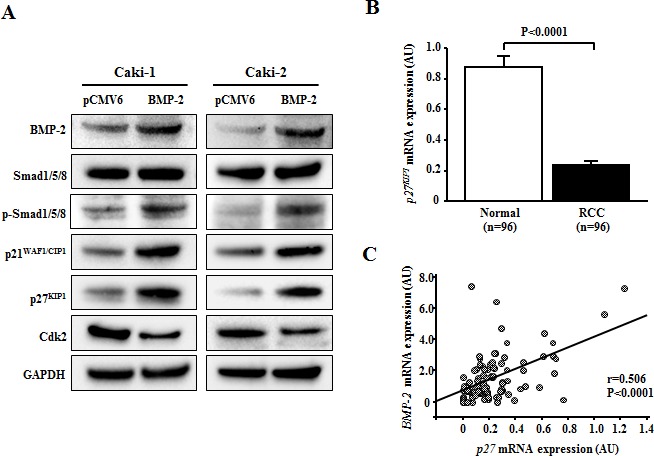
Effect of BMP-2 overexpression on cell cycle regulatory genes (A) Immunoblotting analysis of Smad1/5/8, phospho-Smad1/5/8, p21^WAF1/CIP1^, p27^KIP1^, and Cdk2 in control and BMP-2 transfected Caki-1 and Caki-2 cells. GAPDH was used as a loading control. (B) *p27*^KIP1^ mRNA expression in RCC samples and corresponding normal renal tissues. The *p27*^KIP1^ mRNA transcription was significantly lower in RCC samples than in normal renal tissues (P<0.0001). (C) Relationship of *BMP-2* with *p27*^KIP1^ in RCC samples. A significant inverse correlation was found between *BMP-2* mRNA expression and *p27*^KIP1^ mRNA expression in RCC samples (P<0.0001).

A previous study indicated that the anti-proliferative effect of BMP-2 in RCC cell lines may be due to cell cycle arrest in the G1 phase [18]. In addition, accumulation of p21^WAF1/CIP1^ and/or p27^KIP1^ in response to activation of the BMP-Smad pathway contributes to growth arrest in several types of cancer [22-25]. Therefore we examined the expression of Smad1/5/8, phospho-Smad1/5/8, and several cell cycle regulatory genes using Western analysis. As shown in Fig. 5A, expression of phospho-Smad1/5/8, p21^WAF1/CIP1^, and p27^KIP1^ proteins were significantly increased after transfection with BMP-2 in Caki-1 and Caki-2 cells. Conversely, expression of Cdk2 protein in BMP-2 transfectants in both RCC cell lines was significantly decreased in comparison with the control.

Next, we examined the mRNA expression of *p21^WAF1/CIP1^* and *p27^KIP1^* in clinical samples. That of *p27^KIP1^* was significantly higher in the RCC samples than in the normal renal tissues (P<0.0001; Fig. 5B). Interestingly, there was a significant inverse correlation between *BMP-2* mRNA and *p27^KIP1^* mRNA expression (P<0.0001; Fig. 5C). As for *p21^WAF1/CIP1^*, there was no significant difference in mRNA levels between the RCC samples and normal renal tissues (data not shown).

### BMP-2 effectively induces cellular apoptosis in RCC cells

**Figure 6:**
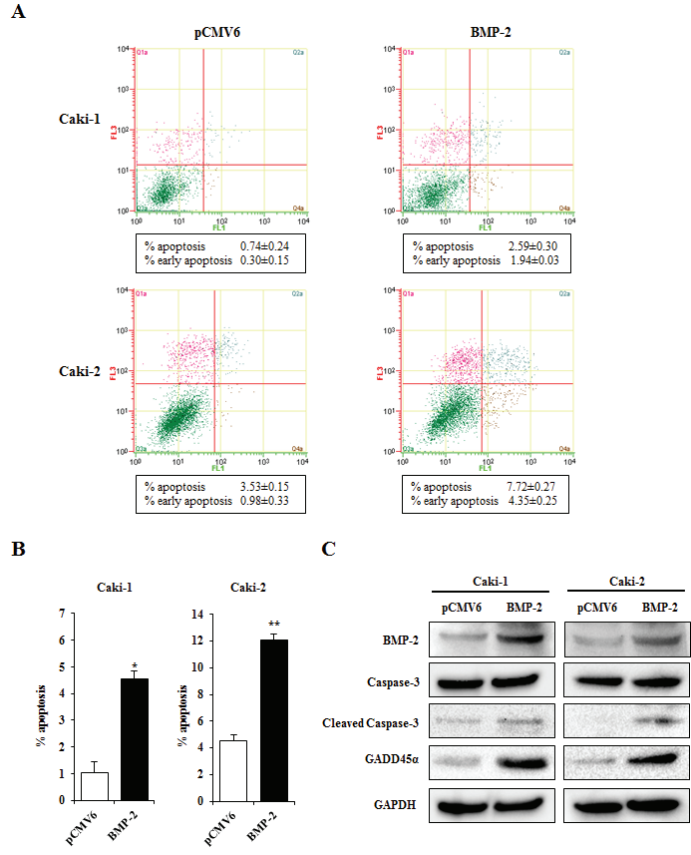
Effects of BMP-2 overexpression on apoptosis (A) Apoptosis assays with Caki-1 and Caki-2 cells were performed at 24 hours after transfection. Representative quadrant figures of control vector BMP-2 transfectants in Caki-1 (upper) and Caki-2 (lower) cells. (B) Bar chart indicates the ratio of apoptotic cell fractions (early plus apoptotic cells) in BMP-2 transfectants as compared with the control. Data for apoptotic cell fractions are expressed as the relative value for the average expression of the control vector transfectant. *P<0.01, **P<0.001. (C) Immunoblotting analysis of apoptotic markers and GADD45β in control and BMP-2 transfected Caki-1 and Caki-2 cells. GAPDH was used as a loading control.

**Figure 7:**
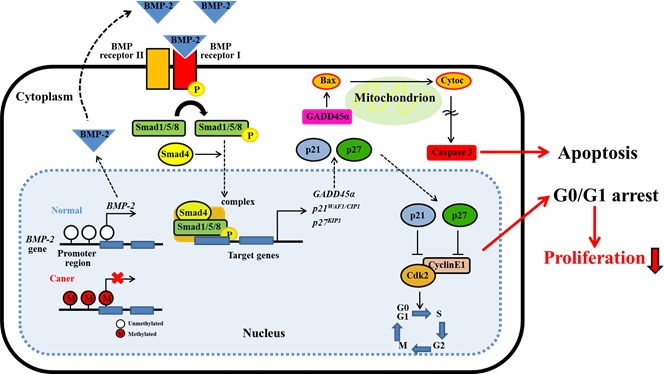
Schema of BMP-2/Smad signaling pathway in RCC BMP-2 expression is regulated by DNA promoter methylation. BMP-2 may cause the induction of GADD45a, p21 and p27 expression through the activation of Smad pathway. GADD45a will induce apoptosis via caspase cascade. In addition, p21 and p27 will induce cell cycle arrest by inhibiting CDK2. These cell cycle arrest and apoptotic effect may play an important role in the inhibition of tumor proliferation in RCC.

Since BMP-2 restoration significantly inhibited proliferation, migration, invasion, and colony formation in RCC cell lines, we hypothesized that its expression may induce apoptosis. The results of apoptosis assays of Caki-1 and Caki-2 cells performed 24 hours post-transfection are shown in Fig. 6A and B. Apoptotic and early apoptotic fractions (upper right and lower right, respectively, in quadrant images) were significantly greater in BMP-2 transfectants as compared to the vector control. These differences were also seen in both Caki-1 and Caki-2 cells at 48 hours after transfection (data not shown). These findings indicate a pro-apoptotic role for BMP-2, as well as its effects on the apoptotic pathway and regulation of tumorigenicity. In our recent study, we found that growth arrest and DNA damage inducible gene 45α (GADD45α) may play important roles in human RCC apoptosis [26]. Therefore, we examined the expression of several apoptotic proteins and GADD45α protein using Western blot analysis. As shown in Fig. 6C, BMP-2 over-expression caused an increase in cleaved caspase-3 in both Caki-1 and Caki-2 cells, further supporting the pro-apoptotic role of BMP-2. Furthermore, GADD45α was up-regulated, indicating that overexpression of BMP-2 induced GADD45α and apoptotic effects. Fig. 7 indicates the putative BMP-2 pathway in RCC on the basis of our results and previous studies [12, 13, 25, 27].

## DISCUSSION

The incidence of RCC has been steadily increasing over recent decades in North America as well as Japan [28, 29]. Although currently employed molecular targeted drugs may be promising as treatment to prevent tumor progression [30, 31], they have not shown robust anti-tumor effects. Therefore, increased understanding of the process of tumor progression may contribute to better treatment options for RCC patients. To date, several studies have shown that BMP-2 inhibits the growth of tumor cells in various types of cancer including RCC [10, 11, 14-18]. With this background in mind, we focused on the role of BMP-2 in RCC tumorigenesis. Our results demonstrated a potential prognostic role for BMP-2 in RCC based on its significantly lower expression in patients with advanced stage and lymph node involvement. We also found that lower OS probability was significantly associated with lower *BMP-2* expression. Furthermore, multivariate analysis clearly demonstrated the prognostic relevance of *BMP-2* expression to predict OS after surgery. Thus, BMP-2 has a strong tumor suppressive potential in human RCC and may be useful for predicting OS following radical nephrectomy.

DNA methylation is one of the most common epigenetic changes that occurs in human cancers and many tumor-related genes are silenced by DNA hypermethylation in RCC [32, 33]. It has been reported that BMP-2 expression is frequently regulated by DNA hypermethylation in colorectal and gastric cancers [14, 15]. Therefore, we evaluated whether BMP-2 expression is regulated by alteration of CpG methylation in RCC. Both Caki-1 and Caki-2 cells showed a significant increase in *BMP-2* mRNA expression following 5 aza-dC treatment compared to non-treated cells and MSP analysis suggested that CpG hypermethylation regulates *BMP-2* gene expression in human RCC cells. We also found that the prevalence of *BMP-2* CpG methylation was significantly higher in RCC samples than normal renal tissues by MSP analysis and bisulfite DNA sequencings. In addition, both BMP-2 mRNA and protein expression levels were significantly lower in methylated than none-methylated RCC samples, indicating epigenetic regulation of BMP-2 in the clinical samples as well. Interestingly, BMP-2 methylation status was correlated with poor prognostic factors (i.e., infiltrative growth pattern and systematic metastasis) and shorter OS as well as BMP-2 expression. This is the first study to report that the expression of BMP-2 is regulated by promoter CpG methylation in RCC.

Overexpression of BMP-2 has been found to decrease cell proliferation, migration, and invasiveness of human colon cancer cell lines [10]. We also noted that BMP-2 re-expression strongly inhibited these activities in RCC cell lines. Previous results indicate that BMP-2 blocs transition of the cell cycle from the G1 to S phase, which then inhibits proliferation of several types of cancer [11, 14, 18]. These anti-proliferative effects seem to be responsible for the increased phosphorylation of Smad1/5/8, and subsequent induction of cyclin-dependent kinase inhibitors such as p21^WAF1/CIP1^ and p27^KIP1^ [22-25]. These two proteins inhibit the activity of the cyclin E/Cdk2 binary complex, which is required for G1/transition [34-36]. Clinically, accumulation of altered cell cycle regulators serves as an independent prognostic factor in RCC [37, 38]. In this study, we demonstrated that the expression of phospo-Smad1/5/8, p21^WAF1/CIP1^, and p27^KIP1^ were up-regulated, while that of Cdk2 was down-regulated after BMP-2 restoration in RCC cell lines. Also, a positive correlation between *BMP-2* and *p27^KIP1^* mRNA levels was found in the RCC samples. These results suggest that BMP-2-induced growth suppression may be partly mediated by induction of p21^WAF1/CIP1^ and/or p27^KIP1^ via activation of a SMAD-dependent signaling pathway in RCC (Fig. 7).

The present study is the first to clearly demonstrate that over expression of BMP-2 induces a significantly increased level of apoptosis in RCC cell lines. Other studies have reported that the anti-proliferative effect of BMP-2 in different tumor cell lines, including gastric, colon, and leukemia, may involve induction of apoptosis [8-10]. However, the precise mechanism of BMP-2-induced apoptosis in cancer cells remains unclear. GADD45 proteins have been implicated in stress response, cell cycle arrest, apoptosis [39, 40], and have also been linked to c-Jun NH (2)-terminal kinase-mediated and mitochondria-mediated cell death [27]. Interestingly *GADD45β*, a member of the GADD45 family, has been proposed to be a major BMP-2 responsive gene induced in chondrocytes [41]. In addition, Tront et al. [42] found that GADD45α may have a tumor suppressive function in breast cancer. We also recently reported that down-regulation of GADD45α inhibits cell death in RCC cell lines [26]. In light of these observations, we sought to determine whether GADD45α is associated with BMP-2-mediated apoptosis and found GADD45α overexpression following BMP-2 restoration in RCC cell lines. Thus, GADD45α may play an essential role in BMP-2-mediated apoptosis in RCC cells (Fig. 7), though further research is required to verify our findings.

In summary, our findings show that BMP-2 is an important tumor suppressor that is down-regulated by promoter CpG hypermethylation in RCC. BMP-2 up-regulates p21^WAF1/CIP1^ and p27^KIP1^ expression and mediates apoptosis causing inhibition of RCC proliferation. Our results strongly suggest that aberrant *BMP-2* methylation and the resultant loss of BMP-2 expression may be useful as a biomarker for designing improved diagnostic and therapeutic strategies for advanced RCC.

## MATERIALS AND METHODS

### RCC tissue samples

Ninety-six matched renal cell carcinoma (90 clear cell carcinomas, 6 papillary carcinomas) and normal renal tissues were obtained from the stocks of Shimane University Hospital (Izumo, Japan). Median patient age at surgery was 63 years old (range 16-87 years). Of the 96 patients, 63 were males and 41 of the lesions were located on the right side. Fifty patients had a stage (Robson's classification) I, 15 patients a stage II, 20 a stage III, and 10 a stage IV. Thirty-three (34.4%) patients had tumor grade 1, 53 (55.6%) were tumor grade 2, and the remaining 10 (10.4%) were tumor grade 3. Of the 96patients, 11 (11.5%) had metastatic disease at the initial diagnosis. Each normal renal and RCC tissue specimen was halved, then one half was fixed in 10% buffered formalin (pH 7.0) and embedded in paraffin wax, with 5-mm-thick sections subjected to H&E staining for histologic evaluation. The remaining half of each sample was immediately frozen and stored at −80°C for DNA and RNA extraction. Written informed consent was obtained from each patient for molecular analysis of the resected specimens and the study protocol was approved by the local ethnical committee of Shimane University Faculty of Medicine.

### Cell lines and reagents

A human renal proximal tubule epithelial cell line, HK-2, and renal cancer cell lines Caki-1 and Caki-2 were obtained from the American Type Culture Collection (Manassas, VA). Keratinocyte serum-free medium (K-SFM), bovine pituitary extract (BPE), and recombinant epidermal growth factor (rEGF) were purchased from Invitrogen (Carlsbad, CA). McCoy's 5A and Opti-minimum essential medium were obtained from the UCSG Cell Culture Facility (San Francisco, CA). Fetal bovine serum (FBS) came from Atlanta Biologicals (Lawrenceville, GA).

### Cell culture

Caki-1 and Caki-2 cells were cultured in McCoy's 5A medium supplemented with 10% FBS. HK-2 cells were maintained in K-SFM supplemented with 0.05 mg/ml BPE and 5 ng/ml rEGF. All cell lines were maintained at 37°C in a humidified atmosphere composed of 5% CO_2_ and 95% air.

### Nucleic acid extraction

Genomic DNA from kidney samples was extracted using a QIAamp Tissue kit (Qiagen, Valencia, CA) and precipitated with ethanol. Genomic DNA from cell lines was extracted using DNAzol reagent (Invitrogen Life Technologies, San Diego, CA) and total RNA was extracted with TRI reagent (Molecular Research Center, Cincinnati, OH), according to the manufacturer's instructions. RNA pellets obtained after isopropanol and ethanol precipitation were dried, resuspended in 25 μL of RNase-free water, and stored in aliquots at −80°C until reverse transcribed. The concentrations of DNA and RNA were determined with a spectrophotometer, and their integrity was checked by gel electrophoresis.

### cDNA preparation and gene quantification

Using 1 μg of RNA, 0.5 μg of oligo-dT primer, and 0.5 units of RNase inhibitor, cDNA was constructed using reverse transcriptase (Promega). The mRNA transcript levels of *BMP-2*, *BMPR1a*, *BMPR1b*, *BMPR2*, *p21^WAF1/CIP1^*, and *p27^KIP1^* were measured with a 7500 Fast Real-Time PCR System (Applied BioSystem), with glyceraldehyde-3-phosphate dehydrogenase (GAPDH) as the reference gene. For clinical samples, a standard curve was generated using a serial dilution of the external standard. The level of expression was calculated as the ratio of the target gene to that of the reference *GAPDH*. For cell line samples, the data was analyzed using the delta-delta Ct method to calculate the fold-change.

### Treatment with 5-aza-2′-deoxycytidine

To screen for epigenetic alterations in the *BMP-2* gene, Caki-1 and Caki-2 cells were treated in duplicate with 5-aza-2′-deoxycytidine (5-aza-dC, 5 μmol/L), then cultured cells were harvested after 4 days of treatment. Using cDNA, the difference in expression level of the *BMP-2* mRNA transcripts before and after 5-aza-dC treatment was analyzed with the 7500 Fast Real-Time PCR System (Applied BioSystem).

### Methylation analysis and bisulfite DNA sequencing

Genomic DNA (100 ng) was modified with sodium bisulfite using a commercial kit (Invitrogen Life Technologies, San Diego, CA). Based on the functional promoter sequence of the *BMP-2* gene [13], methylation- and non-methylation-specific primers were designed using MethPrimer. The regions amplified by these primers have 19 CpG sites and their relationship to the CpG sites are shown in Fig. 2A. for methylation-specific PCR (MSP), a second round of nested PCR (MSP and USP) was done using the universal PCR product amplified by Uni-S and Uni-AS primers as a template. The first universal primer sets had no CpG sites in either the forward or reverse primer. For each assay, the absence of a DNA template served as a negative control. The primer sequences for universal, MSP, and USP are shown in Fig. 2B. The MSP and USP products were analyzed following 2% agarose gel electrophoresis. For bisulfite DNA sequencing, 1 μl of bisulfite-modified DNA was amplified using a pair of universal primers (Uni-S and Uni-AS) in a total volume of 20 μL. Sequencing of the PCR products using either a forward or reverse universal primer was done according to the manufacturer's instructions (Applied Biosystems, Foster City, CA).

### Transfection

For BMP-2 overexpression, Caki-1 and Caki-2 cells were transfected with a pCMV6-ENTRY vector expressing human BMP-2 cDNA or an empty pCMV6-ENTRY vector (OriGene Technologies, Rockville, MD) using X-treme gene HD Transfection Reagent (Roche Diagnostics, Indianapolis, IN), according to the manufacturer's protocol.

### MTS assay

Cells were plated in triplicate in 96-well microplates at a density of 3×10^3^ cells per well, then transfected with BMP-2 the next day. At the desired time points, the number of viable cells was determined by adding a 3-(4,5-dimethylthiazol-2-yl)-5-(3-carboxymethoxyphenyl)-2- (4-sulfophenyl) -2H-tetrazolium-based CellTiter 96 Aqueous One Solution Reagent (Promega, Madison, WI) to each well and measuring absorbance at 490 nm with a SPECTRA MAX 190 plate reader (Molecular Devices, Sunnyvale, CA).

### Migration and invasion assay

Cell migration was evaluated using a wound-healing assay. Cells were plated in 6-well dishes and cell monolayers were scraped using a P-20 micropipette tip. Wound closure was monitored and percent closure measured. Cell invasion assay was performed using modified Boyden Chambers consisting of transwell-pre-coated Matrigel membrane filter inserts with 8 micro-pores in 24-well tissue culture plates (BD Biosciences, Bedford, MA, USA). Transfected cells were re-suspended in culture medium without FBS and placed in the upper chambers in triplicate. After 48 hours of incubation at 37°C, cells migrating through the membrane were stained. The results are expressed as invaded cells quantified at OD 560 nm.

### Colony formation assay

Colony formation assays were performed using a CytoSelect^TM^ 96-Well Cell Transformation Assay (Cell Bioloabs). Briefly, 600 transfected cells were seeded into each well of 96-well microplates and incubated for 8 days. Then, quantitation of colony formation was determined by adding MTT solution to each well and measuring absorbance at 570 nm with a SPECTRA MAX 190 plate reader (Molecular Devices, Sunnyvale, CA).

### Apoptosis assay

Fluorescence-activated cell-sorting (FACS) analysis for apoptosis was done at 24 and 48 hours post-transfection, using an annexin V-fluorescein isothiocyanate (FITC)/7-amino-actinomycin D (7-AAD) staining system (BD Biosciences, San Diego, CA) and a Cell Lab Quanta^TM^ SC MPL (Beckman Coulter, Fullerton, CA). Cells stained with annexin V-FITC only (early apoptotic), or both annexin V-FITC and 7-AAD (late apoptotic) were considered to be apoptotic cell fractions.

### Western blot analysis

Whole cell extracts were prepared using radioimmunoprecipitation assay buffer (RIPA; Thermo Scientific, Rockford, IL) containing a protease inhibitor cocktail (Roche Diagnostics, Basel, Switzerland). Protein quantification was done using a BCA protein assay kit (Pierce) according to the manufacturer's instructions. Total cell protein (15-20 μg) was used for western blotting. Samples were transferred to PVDF membranes, then immersed in 3% skim milk with antibodies against BMP-2 (#ab14933, Abcam, Cambridge, MA), Smad1/5/8 (#12656 Cell Signaling Technology), phospho-SMAD1/5/8 (#sc-12353-R, Santa Cruz), p21^WAF1/CIP1^ (#2947, Cell Signaling Technology), p27^KIP1^ (#2552, Cell Signaling Technology), Cdk2 (#2546, Cell Signaling Technology), Caspase-3 (#9662, Cell Signaling Technology), Cleaved Caspase-3 (#9661, Cell Signaling Technology), and GaDD45α (#4632, Cell Signaling Technology) overnight at 4°C. Blots were washed in TBS containing 0.1% Tween20 and labeled with horseradish peroxidase conjugated secondary anti-rabbit antibody (Cell Signaling Technology). Specific complexes were visualized with an ECHO chemiluminescence (ECL) detection system (GE Healthcare, Little Chalfont, UK) using the Chemidoc imaging system (Bio Rad, CA, USA). The protein expression levels are expressed relative to GAPDH.

### Immunohistochemical analysis

Of the 96 patients with RCC who underwent radical nephrectomy, specimens from 84 were available for evaluation by immunostaining. Normal control tissues were obtained from 62 of those 84 RCC patients. Immunostaining of BMP-2 was performed using an UltraVision Detection System (Thrmo Scientific) according to the manufacturer's instructions. After 12 hours of incubation with a rabbit polyclonal antibody for BMP-2 (1: 250, #ab14933, Abcam), DAB was added as a chromogen, followed by counterstaining with hematoxylin. For BMP-2 expression, cytoplasmic and nuclear expression was analyzed according to the intensity of positive cells using Image J software (http://rsb.info.nih.gov/ij) and ranked on an overall scale from 0 to 3; with 0 indicating the absence of staining; 1, weak staining; 2, moderate staining; and 3, strong staining.

### Statistical analysis

Values are presented as the mean ± standard error based on results obtained from at least 3 independent experiments. All data were analyzed using the StatView V statistical package (SAS Institute, Inc., Cary, NC). Relationships between 2 variables and numerical values were analyzed using a nonparametric Mann-Whitney U test or a two-tailed unpaired Student's *t*-test. A chi-square test was used for analyzing the correlation between clinicopathologic parameters and *BMP-2* methylation status. Correlations between 2 continuous variables were analyzed using Spearman's rank correlation. Survival curves were constructed using the Kaplan-Meir method and differences between 2 curves were analyzed with a log rank test. Univariate and multivariate analyses for overall (OS) survival were performed using a Cox proportional hazards regression model. A P value of less than 0.05 was considered to be statistically significant.

## SUPPLEMENTARY MATERIALS AND FIGURES



## References

[1] Curti BD (2004). Renal cell carcinoma. JAMA.

[2] De Mulder PH, van Herpen CM, Mulders PA (2004). Currentbtreatment of renal cell carcinoma. Annals oncology: official journal of the European Society for Medical Oncology/ESMO.

[3] Siegel R, Naishadham D, Jemal A (2012). Cancer statistics 2012. Cancer J Clin.

[4] Rini BI, Campbell SC, Escudier B (2009). Renal cell carcinoma. Lancet.

[5] Heng DY, Xie W, Regan MM, Harshman LC, Bjarnason GA, Vaishampayan UN, Mackenzie M, Wood L, Donskov F, Tan MH, Rha SY, Agarwal N (2013). External validation and comparison with other models of the International Metastatic Renal-Cell Carcioma Database Consortium prognostic model: a population-based study. Lacet Oncol.

[6] Utsumi T, Ueda T, Fukasawa S, Komaru A, Sazuka T, Kawamura K, Imamoto T, Nihei N, Suzuki H, Ichikawa T (2011). Prognostic models for renal cell carcinoma recurrence: external validation in a Japanese population. Int J Urol.

[7] Wozney JM, Rosen V, Celeste AJ, Mitsock LM, Whitters MJ, Kriz RW, Hewick RM, Wang EA (1988). Novel regulators of bone formation: molecular clones and activities. Science.

[8] Kawamura C, Kizaki M, Ikeda Y (2002). Bone morphogenetic protein (BMP)-2 induces apoptosis in human myeloma cells. Leuk Lymphoma.

[9] Hallahan AR, Pritchard JI, Chandraratna RA, Ellenbogen RG, Geyer JR, Overland RP, Strand AD, Tapscott SJ, Olson JM (2003). BMP-2 mediates retinoid-induced apoptosis in medulloblastoma cells through a paracrine effect. Nat Med.

[10] Zhang Y, Chen X, Qiao M, Zhang BQ, Wang N, Zhang Z, Liao Z, Zeng L, Deng Y, Deng F, Zhang J, Yin L, Liu W (2014). Bone morphogenetic protein 2 inhibits the proliferation and growth of human colorectal cancer cells. Oncol Rep.

[11] Zhang J, Ge Y, Sun L, Cao J, Wu Q, Guo L, Wang Z (2012). Effect of bone morphogenetic protein-2 on proliferation and apoptosis of gastric cancer cells. Int J Med Sci.

[12] Herpin A, Cunningham C (2007). Cross-talk between the bone morphogenetic protein pathway and other major signaling pathways results in tightly regulated cell-specific outcomes. FEBS J.

[13] Nohe A, Keating E, Knaus P, Petersen NO (2004). Signal transduction of bone morphogenetic protein receptors. Cell Signal.

[14] Wen ZX, Akiyama Y, Baylin SB Yuasa Y (2006). Frequent epigenetic silencing of the bone morphogenetic protein 2 gene through methylation in gastric carcinomas. Oncogene.

[15] Kodach LL, Jacobs RJ, Voorneveld PW, Wildenberg ME, Verspaget HW, van Wezel T, Morreau H, Hommes DW, Peppelenbosch MP, van den Brink GR, Hardwick JC (2011). Statins augment the chemosensitivity of colorectal cancer cells inducing epigenetic reprogramming and reducing colorectal cancer cell ‘stemness’ via the bone morphogenetic protein pathway. Gut.

[16] Brubaker KD, Corey E, Brown LG, Vessella RL (2004). Bone morphogenetic protein signaling in prostate cancer cell lines. J Cell Biochem.

[17] Johnsen IK, Kappler R, Auernhammer CJ, Beuschlein F (2009). Bone morphogenetic proteins 2 and 5 are down-regulated in adrenocortical carcinoma and modulate adrenal cell proliferation and steroidogenesis. Cancer Res.

[18] Wang L, Park P, Zhang H, La Marca F, Claeson A, Than K, Rahman S, Lin CY (2012). BMP-2 inhibits tumor growth of human renal cell carcinoma and induces bone formation. Int J Cancer.

[19] Markić D, Ćelić T, Gršković A, Španjol J, Fučkar Ž, Grahovac B, Dorđević G, Bobinac D (2011). mRNA expression of bone morphogenetic proteins their receptors in human renal cell carcinoma. Urol Int.

[20] Sidransky D (2002). Emerging molecular makers of cancer. Nat Rev Cancer.

[21] Kristensen LS, Raynor MP, Candiloro I, Dobrovic A (2012). Methylation profiling of normal individuals mosaic promoter methylation of cancer-associated genes. Oncotarget.

[22] Nakamura Y, Ozaki T, Koseki H, Nakagawara A, Sakiyama S (2003). Accumulation of p27KIP is associated with BMP2-induced growth arrest and neuronal differentiation of human neuroblastoma-derived cell lines. BBRC.

[23] Wu WK, Sung JJ, Wu YC, Li ZJ, Yu L, Cho CH (2008). Bone morphogenetic protein signaling is required for the anti-mitogenic effect of the proteasome inhibitor MG-132 on colon cancer cells. Br J Pharmacol.

[24] Shirai YT, Ehata S, Yashiro M, Yanagihara K, Hirakawa K, Miyazono K (2011). Bone morphogenetic protein-2 and -4 play tumor suppressive roles in human diffuse-type gastric carcinoma. Am J Pathol.

[25] Liu S, Yin F, Fan W, Wang S, Guo XR, Zhang JN, Tian ZM, Fan M (2012). Over-expression of BMPR-IB reduces the malignancy of glioblastoma cells by upregulation of p21 and p27Kip1. J Exp Clin Cancer Res.

[26] Chang I, Liu J, Majid S, Saini S, Zaman MS, Yamamura S, Shahryari V, Chiyomaru T, Deng G, Dahiya R, Tanaka Y (2012). Catechol-O-methyltransferase-mediated metabolism of 4-hydroxyestradiol inhibits the growth of human renal cancer cells through the apoptotic pathway. Carcinogenesis.

[27] Tamura RE, de Vasconcellos JF, Sarkar D, Libermann TA, Fisher PB, Zerbini LF (2012). GADD45 proteins: central players in tumorgenesis. Curr Mol Med.

[28] Simard EP, Ward EM, Siegel R, Jemal A (2012). Cancers with increasing incidence trends in the United States: 1999 through 2008. CA Cancer J Clin.

[29] Washio M, Mori M (2009). Risk factor for renal cell carcinoma in Japanese population. Clin Med Oncol.

[30] Banyra O, Tarchynets M, Shulyak A (2014). Renal cell carcinoma: how to hit the target?. Cent European J Urol.

[31] Belldegrun AS, Chamie K, Kloepfer P, Fall B, Bevan P, Strökel S, Wilhelm O, Pantuck AJ (2013). ARISER: A randomized double blind phase III study to evaluate adjuvant cG250 treatment versus placebo in patients with high-risk ccRCC-Results and implications for adjuvant clinical trials. J Clin Oncol.

[32] Arai E, Kanai Y (2010). Genetic and epigenetic alterations during renal carcinogenesis. Int J Clin Expo Pathol.

[33] Hu CY, Mohtat D, Yu Y, Ko YA, Shenoy N, Bhattacharya S, Izquierdo MC, Park AS, Giricz O, Vallumsetla N, Gundabolu K, Ware K, Bhagat TD (2014). Kidney cancer is characterized by aberrant methylation of tissue-specific enhancers that are prognostic for overall survival. Clin Cancer Res.

[34] Hengst L, Dulic V, Singerland JM, Lees E, Reed SI (1991). A cell cycle-regulated inhibitor of cyclin-dependent kinases. Proc Natl Acad Sci USA.

[35] Polyak K, Lee MH, Erdjument-Bromage H, Koff A, Roberts JM, Tempst P, Massagué J (1994). Cloning p27KIP1, a cyclin-dependent kinase inhibitor and a potential mediator of extracellular antiitogenic signals. Cell.

[36] Toyoshima H, Hunter T (1994). p27, a novel inhibitor of G1 cyclin-Cdk protein kinase activity, is related to p21. Cell.

[37] Sgambato A, Camerini A, Genovese G, De Luca F, Viacava P, Migaldi M, Boninsegna A, Cecchi M, Sepich CA, Rossi G, Arena V, Cittadini A, Amoroso D (2010). Loss of nulear p27KIP1 and α-dystroglycan is a frequent event and is a strong predictor of poor outcome in renal cell carcinoma. Cancer Sci.

[38] Gayed BA, Youssef RF, Bagrodia A, Kapur P, Darwish OM, Krabbe LM, Sagalowsky A, Lotan Y, Margulis V (2013). Prognostic role of cell cycle and proliferative biomarkers in patients with clear cell renal cell carcinoma. J Urol.

[39] Abdollahi A, Lord KA, Hoffman-Liebermann B, Liebermann DA (1991). Sequence and expression of a cDNA encoding MyD118: a novel myeloid differentiation primary response gene induced by multiple cytokines. Oncogene.

[40] Amanullah A, Azam N, Balliet A, Hollander C, Hoffman B, Fornace A, Liebermann D (2003). Cell signalling: cell survival and a Gadd45-factor deficiency. Nature.

[41] Ijiri K, Zerbini LF, Peng H, Correa RG, Lu B, Walsh N, Zhao Y, Taniguchi N, Huang XL, Otu H, Wang H, Wang JF, Komiya S (2005). A novel role for GADD45beta as a mediator of MMP-13 gene expression during chondrocyte terminal differentiation. J Biol Chem.

[42] Tront J, Hoffman B, Liebermann D (2006). Gadd45a suppresses ras-driven mammary tumorigenesis by activation of JNK and p38 stress signaling resulting in apoptosis and senescence. Cancer Res.

