# Supplementary material for: Inactivation of bone morphogenetic protein 2 may predict clinical outcome and poor overall survival for renal cell carcinoma through epigenetic pathways
Source: Oncotarget. 2015 Mar 7;6(11):9577–91. doi: 10.18632/oncotarget.3445 (PMC4496240; doi:10.18632/oncotarget.3445)
Supplement: Supplementary file 1 [file oncotarget-06-9577-s001.pdf]

# Inactivation of bone morphogenetic protein 2 may predict clinical outcome and poor overall survival for renal cell carcinoma through epigenetic pathways

**Supplementary Material**

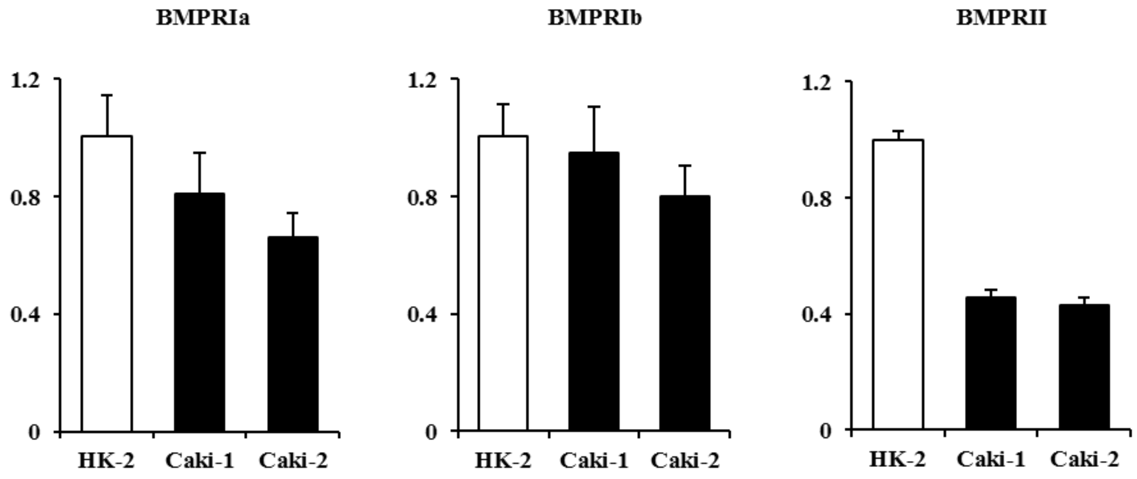


**Figure S1: mRNA expression of BMP-2 receptors in HK-2 and RCC cell lines.**

BMPRIa, BMPRIb, and BMPRII were found to be expressed in Caki-1 and Caki-2 cells, with significantly lower levels of BMPRII as compared to HK-2 cells. *P<0.05, **P<0.01.
